# Molecular Adaptation of *rbcL* in the Heterophyllous Aquatic Plant *Potamogeton*

**DOI:** 10.1371/journal.pone.0004633

**Published:** 2009-02-27

**Authors:** Satoko Iida, Atsuko Miyagi, Seishiro Aoki, Motomi Ito, Yasuro Kadono, Keiko Kosuge

**Affiliations:** 1 Research Center for Environmental Genomics, Kobe University, Kobe, Hyogo, Japan; 2 Department of Biology, Faculty of Science, Kobe University, Kobe, Hyogo, Japan; 3 Graduate School of Arts and Sciences, The University of Tokyo, Tokyo, Japan; University of Exeter, United Kingdom

## Abstract

**Background:**

Heterophyllous aquatic plants show marked phenotypic plasticity. They adapt to environmental changes by producing different leaf types: submerged, floating and terrestrial leaves. By contrast, homophyllous plants produce only submerged leaves and grow entirely underwater. Heterophylly and submerged homophylly evolved under selective pressure modifying the species-specific optima for photosynthesis, but little is known about the evolutionary outcome of habit. Recent evolutionary analyses suggested that *rbcL*, a chloroplast gene that encodes a catalytic subunit of RuBisCO, evolves under positive selection in most land plant lineages. To examine the adaptive evolutionary process linked to heterophylly or homophylly, we analyzed positive selection in the *rbcL* sequences of ecologically diverse aquatic plants, Japanese *Potamogeton*.

**Principal Findings:**

Phylogenetic and maximum likelihood analyses of codon substitution models indicated that *Potamogeton rbcL* has evolved under positive Darwinian selection. The positive selection has operated specifically in heterophyllous lineages but not in homophyllous ones in the branch-site models. This suggests that the selective pressure on this chloroplast gene was higher for heterophyllous lineages than for homophyllous lineages. The replacement of 12 amino acids occurred at structurally important sites in the quaternary structure of RbcL, two of which (residue 225 and 281) were identified as potentially under positive selection.

**Conclusions/Significance:**

Our analysis did not show an exact relationship between the amino acid replacements and heterophylly or homophylly but revealed that lineage-specific positive selection acted on the *Potamogeton rbcL*. The contrasting ecological conditions between heterophyllous and homophyllous plants have imposed different selective pressures on the photosynthetic system. The increased amino acid replacement in RbcL may reflect the continuous fine-tuning of RuBisCO under varying ecological conditions.

## Introduction

Many aquatic plants exhibit marked developmental plasticity, known as heterophylly. Heterophylly occurs widely across distantly related taxa and is thought to have arisen via convergent evolution [1]. Heterophyllous species have broad, thick leaves with stomata on the water surface (floating leaves), as well as elongated, thin leaves without stomata within the water column growing from submerged shoots (submerged leaves). In addition, they sometimes produce terrestrial leaves from land shoots during droughts. In contrast, homophyllous aquatic plants produce only submerged leaves and grow entirely underwater. Each leaf type appears to be adapted to its environment morphologically and anatomically [2]–[4]. Moreover, the submerged and floating leaves have different photosynthetic properties [5]. The degree of heterophylly (the shapes of the submerged, floating, and terrestrial leaves) varies from population to population, depending on the water level fluctuation in each habitat [6], [7]. Therefore, heterophylly and submerged homophylly evolved under selective pressure modifying the species-specific optima for photosynthesis.

*Potamogeton* L. (Potamogetonaceae), one of the largest genera of aquatic angiosperms, is ecologically diverse and distributed in various freshwater bodies (lakes, marshes, rivers, artificial ponds, etc.), as well as in brackish water [4], [8]. The genus includes both heterophyllous and homophyllous (strictly submerged) species. Recent molecular phylogenetic analyses of *Potamogeton* revealed that homophylly is the ancestral state and heterophylly has evolved several times in different lineages, possibly due to parallel evolution [9], [10]. Furthermore, we have reported that the degree of heterophylly in natural hybrids depends on the maternal type in reciprocal crosses [11]. The parental taxa of the hybrid are closely related but differ ecologically; *P. malaianus* Miq. can survive droughts by producing terrestrial shoots and is frequently distributed in shallow and inshore areas; *P. perfoliatus* L. generally lacks such phenotypic plasticity, grows in deeper water, and is more shade tolerant. Despite the high similarity of the *trnT*-*trnL* intergenic region [9], all three substitutions between *rbcL* of the parental species are nonsynonymous [11]. Even single amino acid replacements in RbcL could account for differences in the CO_2_ and O_2_ specificity of ribulose 1,5-bisphosphate carboxylase/oxygenase (RuBisCO) [12]. The maternal effect on survival under drought stress and the depth distribution of reciprocal hybrids [11] has led to speculation that the amino acid difference in RbcL plays an important role in ecological adaptation.

RbcL provides all the catalytically essential residues of RuBisCO, a critical enzyme for both the reductive and oxidative photosynthetic carbon cycles. The activity of RuBisCO is thought to be limited by environmental stresses, such as oxidative stress, heat, osmotic stress, and drought [13]–[15]. The holoenzyme consists of eight large catalytic subunits (RbcLs) and eight small subunits (RbcSs). The sequence of *rbcL* has great phylogenetic utility because of its conserved nature [16], although substitutions occur in sites of known functional importance [17], [18]. The amino acid replacements in RbcL at the interface of subunits are correlated with the loss and gain of pyrenoids in the unicellular green alga lineage [19]. In addition, positive Darwinian selection of *rbcL* has been detected in cyanobacteria [20] and various taxonomic groups of land plants [21]–[23].

*Potamogeton* is a representative example of the adaptive evolution of heterophylly. Japanese *Potamogeton* taxa are highly diversified in growth forms and leaf types and represent 11 of the 14 species groups in the genus *Potamogeton* [9], [24]. In this study, to gain insight into whether an adaptive evolutionary process is linked to heterophylly or homophylly in Japanese *Potamogeton*, we tested whether there was positive selection in *rbcL* using phylogenetic and maximum likelihood analyses of codon substitution models [25]–[27]. We demonstrated that the evolution of ecologically divergent Japanese *Potamogeton* has involved the molecular adaptation of *rbcL*. In this article, abstract is also available in Japanese (Text S1).

## Results

### Evidence for positive selection in *Potamogeton rbcL*

**Figure 1:**
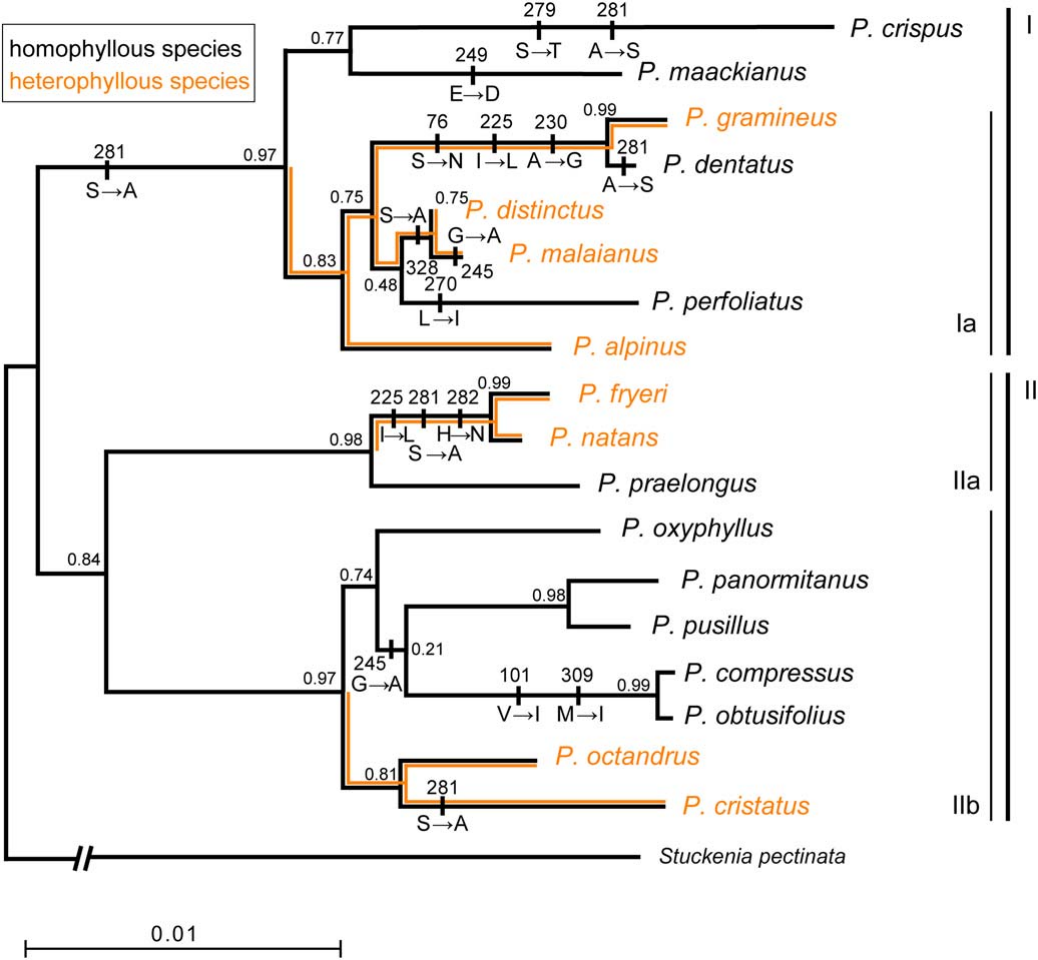
Maximum likelihood *Potamogeton* cpDNA tree based on a combined 4394-bp chloroplast DNA dataset. Approximate likelihood-ratio test measures (aLRT) are indicated as support for branches [52]. Amino acid replacements of RbcL reconstructed under the codon substitution model (M8) are also indicated. Branches with orange lines indicate three heterophyllous lineages, whereas the others were homophyllous lineages.

Three genes (*rbcL*, *atpB*, *petA*) were sequenced from 18 Japanese *Potamogeton*. All the nucleotide sequences determined in this study were deposited in GenBank (Table S1). The phylogenetic trees based on individual chloroplast genes were consistent with each other and with a previous phylogenetic study [9]. A robust gene tree of chloroplast DNA is essential to conduct a maximum likelihood analysis in the molecular adaptation test. We combined all four datasets (1349 *rbcL*, 1467 *atpB*, 918 *petA*, and 660 *trnT*-*trnL* intergenic spacer sites) to obtain a well-resolved gene tree of the *Potamogeton* chloroplast DNA (cpDNA tree; Fig. 1). The basal branches of the cpDNA tree were congruent with those found in a previous study of the *trnT*-*trnL* intergenic spacer [9]. The Japanese *Potamogeton* species were divided into group I, with broad submerged leaves, and group II, with linear submerged leaves and subepidermal bundles. The latter was further divided into subgroups IIa and IIb (Fig. 1). In addition, newly resolved terminal branches identified several species groups (Fig. 1), each of which was characterized by morphological features [9], [28], [29]. For example, subgroup Ia (Fig. 1) was characterized by the presence of overwintering subterranean turions.

To test the hypothesis of positive selection in *rbcL*, we examined the fit of the *Potamogeton rbcL* sequences to codon substitution models based on the cpDNA tree (Fig. 1). Table 1 lists the parameter estimates and log-likelihood values for the eight codon substitution models of molecular evolution. In order to evaluate positive selection, we performed four likelihood ratio tests comparing M1A (nearly neutral) with M2A (positive selection), M7 (beta) with M8 (beta and ω_s_≥1), M8A (beta and ω_s_ = 1) with M8 (beta and ω_s_≥1), and branch-site model A (ω_2_ = 1 fixed) with branch-site model A (ω_2_ estimated) (Table 2).

**Table 1:** Parameter estimates and log-likelihood values for *Potamogeton rbcL* under eight codon substitution models included in PAML.

Model	Log-likelihood	Parameters a
**Site-specific models**		
M0 : one ω	−2182.1	ω = 0.129
M1A : nearly neutral	−2167.3	*p_0_* a = 0.912, ω_0_ = 0.000
		*p_1_* = 0.088, ω_1_ = 1.000
M2A : positive selection	−2163.1	*p_0_* = 0.914, ω_0_ = 0.000
		*p_1_* = 0.082, ω_1_ = 1.000
		*p_2_* = 0.004, ω_2_ = 11.494
M7 : beta	−2167.4	*p* = 0.005, *q* = 0.0482
M8 : beta & ω_s_≥1	−2163.4	*p_0_* = 0.963
		*p* = 0.010, *q* = 0.314
		*p_1_* = 0.037, ω_1_ = 3.825
M8A : beta & ω_s_ = 1	−2167.3	*p_0_* = 0.912
		*p* = 0.005, *q* = 99.000
		*p_1_* = 0.088, ω_1_ = 1.000
**Branch-site models**		
Foreground: Heterophylly		
Model A (ω_2_ = 1 fixed)	−2166.7	*p_0_* = 0.871, ω_0_ = 0.000
		*p_1_* = 0.064, ω_1_ = 1.000
		*p_2+_p_3_* = 0.065 , ω_2_ = 1.000
Model A (ω_2_ estimated)	−2162.1	*p_0_* = 0.920, ω_0_ = 0.000
		*p_1_* = 0.066, ω_1_ = 1.000
		*p_2+_p_3_* = 0.013 , ω_2_ = 26.150
Foreground: Homophylly		
Model A (ω_2_ = 1 fixed)	−2166.0	*p_0_* = 0.850, ω_0_ = 0.000
		*p_1_* = 0.053, ω_1_ = 1.000
		*p_2+_p_3_* = 0.097 , ω_2_ = 1.000
Model A (ω_2_ estimated)	−2165.8	*p_0_* = 0.899, ω_0_ = 0.000
		*p_1_* = 0.055, ω_1_ = 1.000
		*p_2+_p_3_* = 0.046 , ω_2_ = 2.575

a the proportion (*p_i_*) of codon sites with ω_i_. In models M7, M8 and M8A, ω was drawn from a beta distribution B(*p*, *q*) for a proportion (*p_0_*) of sites.

**Table 2:** LRT statistics for testing the hypothesis of positive selection in *Potamogeton rbcL*.

Test No.	Test model	Null model	df	2ΔL^b^
Test 1	M2A	M1A	2	8.4*
Test 2	M8	M7	2	8.0*
Test 3	M8	M8A	-a	7.8**
Test 4	Branch-site model A (ω_2_ estimated)	Branch-site model A (ω_2_ = 1 fixed)		
		Foreground: Heterophylly	1	9.2**
		Foreground: Homophylly	1	0.2

a The test statistic for the M8A-M8 comparison is compared with 50∶50 mixture of df = 0 and 1 according to Swansson et al. (2003).

b *: *p*<0.05, **: *p*<0.01.

The average ω ratio of *Potamogeton rbcL* was 0.129 over all 449 codon sites and lineages (Table 1, M0). Assuming that the ω ratio of all sites was either ω = 0 or ω = 1 and for the average of all the lineages in the tree, a majority of the sites (91.2%) were under purifying selection with ω = 0 in model M1A (Table 1). The remaining sites (8.8%) were under neutral evolution with ω = 1, indicating the dominant role of purifying selection in the evolution of *rbcL*. In model M2A, the majority of the sites (91.4%) were under purifying selection, 8.2% of the sites were under neutral evolution, and 0.4% of the sites experienced positive selection (ω = 11.494)(Table 1). The likelihood ratio test revealed that model M2A fit the data significantly better than the null model M1A (Table 2).

Model M7 assumed several codon site classes with values of ω ranging from 0 to 1 (Table 1). By contrast, 3.7% of the codons had ω = 3.825 and experienced positive selection in model M8 (Table 1). Once again, the *Potamogeton rbcL* sequences fit model M8, which allowed positive selection better than the null model M7 (Table 2).

In Model M8A, an additional category of sites with ω = 1 was added to the null model M7 (Table 1). The hypothesis of positive selection on *Potamogeton rbcL* was also supported by a test comparing M8 against the null model M8A (Table 2), which may be more robust than M1A *vs.* M2A or M7 *vs.* M8 [30].

The branch-site models allow the ω ratio to vary both among codon sites and among lineages. Heterophyllous lineages in the *Potamogeton* cpDNA tree were specified *a priori* as foreground branches that might be expected to be under positive selection (Fig. 1). In model A (ω_2_ estimated), a majority of the sites were under purifying selection (92.0%), whereas 6.6% of the codons were evolving neutrally throughout the tree. The remaining sites (1.34%) were either purifying (1.25%) or neutral (0.9%) on the background branches, but came under positive selection on the foreground branches with ω = 26.150 (Table 1). Test model A (ω_2_ estimated) fit *Potamogeton rbc*L significantly better than null model A (ω_2_ = 1 fixed; Table 1)(Table 2). Conversely, when homophyllous lineages within the genus *Potamogeton* were specified as foreground branches, both the test and null models had similar log-likelihood values (Tables 1 and 2).

The other chloroplast encoded genes, *atpB* and *petA*, have reported evolutionary rates similar to *rbcL* [31]. The average ω ratios of *Potamogeton atpB* and *petA* were 0.098 and 0.174, respectively (Tables S3 and S4). In both genes, positive selection was detected in the site-specific models analyses with a high posterior probability of evolving under positive selection for two *atpB* (sites 16 and 488) and one *petA* (site 239) residues but not in the branch-site models (Tables S3 to S5).

### Features of amino acid replacement sites

Table 3 shows the distribution of 12 variable amino acid sites in RbcLs from *Potamogeton* and the outgroup species. Amino acid replacements at residues 225 and 281 were identified as potentially under positive selection. Most of the replacements were implied to have occurred at terminal branches of the phylogenic tree, rather than at basal ones (Fig. 1). The replacement of residue 225 (Ile^225^→Leu^225^, codon exchange ATT→CTT) was thought to have occurred twice: once in subgroup Ia and again in subgroup IIa. Residue 281 was replaced five times: three parallel changes of Ser^281^→Ala^281^ (TCT→GCT) and two of Ala^281^→Ser^281^ (GCT→TCT).

**Table 3:** Amino acid replacement sites in 18 *Potamogeton* species and *Stuckenia* RbcL.

Amino acid site	76	101	225	230	245	249	270	279	281	282	309	328
Species												
*P. crispus* (−a)	S	V	I	A	G	E	L	T	S	H	M	S
*P. maackianus* (−a)	S	V	I	A	G	D	L	S	A	H	M	S
*P. gramineus* (++)	N	V	L	G	G	E	L	S	A	H	M	S
*P. dentatus* (−)	N	V	L	G	G	E	L	S	S	H	M	S
*P. distinctus* (++)	S	V	I	A	G	E	L	S	A	H	M	A
*P. malaianus* (++)	S	V	I	A	A	E	L	S	A	H	M	A
*P. perfoliatus* (−a)	S	V	I	A	G	E	I	S	A	H	M	S
*P. alpinus* (+), *P. cristatus* (+)	S	V	I	A	G	E	L	S	A	H	M	S
*P. fryeri* (++), *P. natans* (++)	S	V	L	A	G	E	L	S	A	N	M	S
*P. praelongus* (−), *P. oxyphyllus* (−), *P. octandrus* (+)	S	V	I	A	G	E	L	S	S	H	M	S
*P. compressus* (−), *P. obtusifolius* (−)	S	I	I	A	A	E	L	S	S	H	I	S
*P. panormitanus* (−a), *P. pusillus* (−)	S	V	I	A	A	E	L	S	S	H	M	S
*Stuckenia pectinata* (−a)	S	V	I	A	G	E	L	S	S	Y	M	S
Structural feature^b^	i	c	c	i	D	D	*c	D	c	D	D	*c
Posterior probabilities of ω≥1^c^												
Site-specific model (M8)	.51	.38	.86	.48	.85	.32	.49	.48	**1.0**	.86	.43	.38
Branch-site model A (estimated ω_2_, Foreground: Heterophylly)	.73	.02	**.98**	.74	.29	.02	.72	.03	**.98**	.29	.02	.68

Number corresponds to the amino acid position of spinach RbcL. (−): homophyllous plant with submerged leaves, (+): heterophyllous plant with submerged and floating leaves, (++): heterophyllous plant with submerged, floating and terrestrial leaves.

a the species is also distributed in brackish water.

b *: residues that are close to the active sites, c: residues that are buried in the interior of RbcL, D: residues at the intradimer interface of RbcL, i: residues at the interface between RbcL and RbcS.

c probabilities above 0.95 are indicated in bold.

In order to infer the possible effects of amino acid replacement sites, the structural motif of *Spinacia oleracea* RbcL [32] was used (1UPP and 1RCX were obtained from the RCSB Protein Data Bank) (Table 3). Many amino acid replacements in *Potamogeton* RbcLs have occurred at the interface between subunits and near the active sites. Seven amino acids (76, 230, 245, 249, 279, 282, and 309) were located at the surface of the RbcL molecule. Two of them (76 and 230) were at the interface between RbcL and RbcS, whereas five (245, 249, 279, 282, and 309) were at the interface of the RbcL dimer. The remaining four (101, 225, 281, and 328) were buried within the molecule. Residue 270 was also located within the molecule, although it was not completely buried. Residues 270 and 328 were close to some of the active sites (Arg^295^, His^298^, and His^327^) in substrate-binding regions [32].

## Discussion

In *Potamogeton*, heterophyllous species are distributed in freshwater and sometimes on the shore, whereas homophyllous species live entirely underwater in fresh or brackish water. The contrasting ecological conditions between heterophyllous and homophyllous plants may have imposed different selective pressures on the photosynthetic system. Our molecular evolutionary analyses indicated that *Potamogeton rbcL* has evolved under positive selection (Tables 1 and 2). Of 12 amino acid sites, two (sites 225 and 281) were potentially driven by positive selection (Table 3). The replacement at site 281 appeared to have originated independently in different phylogenetic lineages of both heterophyllous and homophyllous species (Fig. 1).

Contrary to our observations, positive selection was absent in the *rbcL* of two exclusively aquatic lineages: monocots-4 (Potamogetonaceae plus Zosteraceae) and monocots-9 (Hydrocharitaceae) [22]. Sample size may account for the differences. Compared to the enormous numbers and variety of land plant *rbcL*, nonsynonymous substitutions in each aquatic lineage were limited, so weak signals of positive selection could not be detected. Indeed, the presence of positive selection was shown in a combined *rbcL* dataset for both lineages [22]. The ω ( = *dN*/*dS*) ratio in *Potamogeton rbcL* (0.129) was approximately twice that found in seagrass *Zostera* (0.050). Ecological differences between aquatic and terrestrial habitats may account for this increase in nonsynonymous substitution in *Potamogeton rbcL*.

Most aquatic plants are thought to have originally thrived on land and adapted secondarily to an aquatic habitat. Therefore, the heterophyllous condition arose as the land became submerged. On the other hand, the phylogenetic tree of Alismatales [33] (Angiosperm Phylogeny Website, Version 9, June 2008 by Stevens PF [http://www.mobot.org/MOBOT/research/APweb/]) suggests that the Potamogetonaceae and its sister family Zosteraceae originated entirely from submerged seagrasses. Considering such secondary adaptation to the terrestrial environment, the heterophyllous taxa of *Potamogeton* are surmised to have been derived from homophyllous taxa.

Terrestrial plants are commonly exposed to temperatures of 40 to 50°C for several hours a day [34]. Heterophyllous taxa usually produce floating or terrestrial leaves in the summer [3], [35]. The temperature of the floating leaves can exceed 30°C [36]. To improve their intrinsic thermal stability, proteins have more hydrophobic residues with branched side chains and more charged residues, at the expense of uncharged polar residues [37]. Molecular evolutionary analysis of cyanobacteria RbcL suggests that adaptive replacement, an increase in hydrophobic residues and a decrease in uncharged polar residues, occurred in a clade of hot spring strains [20]. In *Potamogeton* RbcL, the uncharged polar residue Ser was replaced with the hydrophobic residue Ala at site 281 (Table 3). This amino acid replacement might increase the thermal stability of RbcL in the heterophyllous taxa. However, recent evolutionary analyses of RuBisCO suggested that amino acid site 281 underwent parallel genetic changes; the Ala in almost all C_3_ photosynthesis species was replaced by Ser in the C_4_ group [23]. The replacement of Ala^281^ by Ser^281^ occurred in two homophyllous species (*P. crispus* and *P. dentatus*), and the reverse from Ser^281^ to Ala^281^ occurred in two independent heterophyllous lineages (Fig. 1). A significant correlation was found between the presence or absence of heterophylly and the amino acid at residue 281 (*p*<0.05 by Fisher's exact test). The detection of positive selection in heterophyllous lineages suggested that selective pressure on this chloroplast gene was higher for heterophyllous lineages than for homophyllous lineages. In the evolutionary analyses of *rbcL* sequences from over 3000 species representing green plants and other phototroph lineages, the neighboring residues 279 and 282 in helix 4 were identified as evolving under positive selection [22]. This suggests that a replacement at these amino acid sites could be involved in the adaptation of specificity or catalytic efficiency to widespread environmental variation in temperature and dryness in both terrestrial land plants (C_4_ and C_3_ plants) and *Potamogeton*.

Many aquatic plants have acquired carbon-concentrating mechanisms to overcome the potentially low, fluctuating supply of CO_2_ for underwater photosynthesis [38]. A strategy of C_4_-like carbon fixation operates in several freshwater homophyllous species [38]–[40] and seagrasses [41]. The evolutionary analysis of the RuBisCO gene revealed that five amino acid sites in *rbcL* evolved under positive selection in terrestrial C_4_ monocots [23]. At three amino acid sites, residues of the C_4_-like seagrass *Zostera* were fixed and shared with those of terrestrial C_4_ monocots (Ile^101^, Ile^270^, and Ser^281^)[42]. In *Potamogeton*, some homophyllous plants share residues with C_4_ monocots (Ile^101^, Ile^270^, Ser^281^, and Ile^309^; Table 3). The homophyllous species *P. perfoliatus*, which is located terminally in the cpDNA tree, had Ile^270^ (Fig. 1, Table 3). This plant tends to be shade tolerant and grows in deep fresh water and brackish water [11]. Its sister species *P. malaianus* is heterophyllous, shade intolerant, and distributed mainly in shallow, inshore freshwater [11]. Our ongoing studies indicate that underwater heat stress induces the transcription of key enzymes in C_4_ photosynthesis (phosphoenolpyruvate carboxylase and pyruvate, orthophosphate dikinase) in submerged leaves of *P. perfoliatus* but not in those of *P. malaianus*. This suggests that amino acid replacement in *P. perfoliatus* RbcL could be involved in acquiring C_4_-like photosynthesis. However, the selective pressure involved appeared to be mild, as no positive selection specific to homophyllous lineages was detected.

### Conclusion

Our analysis did not show an exact relationship between the amino acid replacements and heterophylly or homophylly but revealed that positive selection has affected the *Potamogeton rbcL*. Polyploidy and aneuploidy, major forces in diversification, are common within the genus [43]. We are now conducting phylogenetic analyses of four nuclear genes to infer the polyploid history of Japanese *Potamogeton*. Our preliminary data suggest that heterophylly has evolved through allopolyploid formation. The increased amino acid replacement in *rbcL* may reflect the adaptive fine-tuning of RuBisCO in the alternation of growth forms (heterophyllous or homophyllous) and nuclear genome setsz.

## Materials and Methods

Japanese *Potamogeton* species and the allied species *Stuckenia pectinata* (L.) Börner were used in the analyses (Table S1). Total DNA was prepared previously for a phylogenetic analysis of Japanese *Potamogeton* based on the *trnT*-*trnL* region [9]. We categorized the *Potamogeton* species into three groups based on growth form and leaf type: homophyllous species (submerged leaves), floating-leaved heterophyllous species (submerged and floating leaves), and terrestrial-leaved heterophyllous species (submerged, floating, and terrestrial leaves), according to the descriptions of Kadono (1984) [44]. The production of terrestrial leaves has been observed in some homophyllous species (*P. dentatus* and *P. perfoliatus*) under cultivation or on a few anomalous occasions in the field [11], [44]–[46]. Nevertheless these species were treated as homophyllous, as their terrestrial leaves did not seem to contribute to persistent survival.

### Amplification and sequencing of chloroplast-encoded genes

The amplification and sequence analysis of chloroplast-encoded genes (*rbcL*, *atpB*, and *petA*) were performed as described previously [9]. Twenty-five PCR cycles at 94°C for 30 s, 59°C for 40 s, and 72°C for 60 s were performed. The forward and reverse primers used for amplification are listed in Table S2.

### Molecular evolutionary analysis

Maximum likelihood (ML) trees were produced online (http://www.atgc-montpellier.fr/phyml/) using the program PhyML [47]. Five starting trees (neighbor-joining and four equally parsimonious trees) were examined with the substitution model HKY85, and the phylogenetic tree with the largest likelihood was selected. *Stuckenia pectinata* was used as the outgroup. Two length mutations (a 9-bp deletion in *P. gramineus* and *P. dentatus atpB* and a 6-bp insertion in *P. praelongus petA*) were treated as missing data and excluded from the analysis.

Molecular adaptation tests on the *Potamogeton rbcL* codon sites and reconstruction of the ancestral *rbcL* sequences were performed using maximum-likelihood models and programs included in PAML ver. 4.1 [25]–[27], [48], [49]. The models used the nonsynonymous/synonymous substitution rate ratio (ω = *dN*/*dS*) as an indicator of selective pressure and allowed the ratio to vary among codon sites. We used five site-specific codon substitution models: null models for testing positive selection (M1A, M7, and M8A) and models allowing for positive selection (M2A and M8) [50], [51]. The likelihood ratio test was used to compare these alternative models. Using model 0 included in the same program, the average nonsynonymous/synonymous substitution rate ratio (ω = *dN*/*dS*) was also estimated.

In contrast to the site-specific models, which assume the same ω ratio among all of the lineages in the tree, the branch-site models let the ω ratio vary among both sites and lineages [26], [27]. To clarify whether positive selection is linked to the evolution of heterophylly or homophylly, the branch-site models implemented in PAML ver. 4.1 (the modified branch-site model A) [27], [48] used heterophylly or homophylly to define branches tested for positive selection (foreground branches). As a single origin of heterophylly is more parsimonious than multiple origins, subgroup Ia was assigned as a heterophyllous lineage for the selection test, in addition to the other monophyletic lineages of heterophyllous species (Fig. 1). Such a definition of heterophyllous lineage was considered logical, as the homophyllous species included in subgroup Ia (*P. dentatus* and *P. perfoliatus*) has been observed to produce anomalous terrestrial leaves [11], [44]–[46].

The posterior probabilities that a site was drawn from a given ω site class were calculated using the parameter estimates from model M8 or branch-site model A (ω_2_ estimated, foreground: heterophylly) and a procedure called the Bayes empirical Bayes method [51]. A site was considered potentially under positive selection when it had probabilities above 0.95. All the above molecular evolutionary analyses were also carried out for the other chloroplast genes, *atpB* and *petA*.

To identify the amino acid position for the structural motif, the quaternary structure of RbcL was inferred from data for *Spinacia oleracea* L. [32] (1UPP and 1RCX obtained from the RCSB Protein Data Bank: http://www.rcsb.org/pdb).

## Supporting Information

Table S1 List of *Potamogeton* and *Stuckenia* species with GenBank accession numbers of chloroplast genes examined in the present study.
(0.05 MB DOC)

Table S2 List of primers used for PCR.
(0.03 MB DOC)

Table S3 Parameter estimates and log-likelihood values for *Potamogeton atpB* under eight codon substitution models included in PAML.
(0.05 MB DOC)

Table S4 Parameter estimates and log-likelihood values for *Potamogeton petA* under eight codon substitution models included in PAML.
(0.05 MB DOC)

Table S5 LRT statistics for testing the hypothesis of positive selection in *Potamogeton* chloroplast genes.
(0.05 MB DOC)

Text S1 Abstract in Japanese.
(0.03 MB DOC)
