# Supplementary material for: Molecular Adaptation of rbcL in the Heterophyllous Aquatic Plant Potamogeton
Source: PLoS One. 2009 Feb 27;4(2):e4633. doi: 10.1371/journal.pone.0004633 (PMC2646136; doi:10.1371/journal.pone.0004633)
Supplement: Table S1 — List of Potamogeton and Stuckenia species with GenBank accession numbers of chloroplast genes examined in the present study. (0.05 MB DOC) [file pone.0004633.s001.doc]

**Table S1** List of *Potamogeton* and *Stuckenia* species with GenBank accession numbers of chloroplast genes examined in

the present study.

| Species | Species group^a^ | Accession No. | | | |
| --- | --- | --- | --- | --- | --- |
|  |  | *trnT-trnL* | *rbcL* | *atpB* | *petA* |
| *P. alpinus* L. | Polygonifolius | AB120537 | AB196845 | AB250110 | AB250129 |
| *P. distinctus* A. Benn. | Nodosus | AB120553 | AB196941 | AB250111 | AB250130 |
| *P. fryeri* A. Benn. | Amplyfolius | AB120538 | AB196942 | AB250112 | AB250131 |
| *P. natans* L. | Natans | AB120540 | AB196946 | AB250113 | AB250132 |
| *P. dentatus* Hagstr. | Lucens | AB120539 | AB196940 | AB250114 | AB250133 |
| *P. gramineus* L. | Lucens | AB120554 | AB196943 | AB250115 | AB250134 |
| *P. malaianus* Miq. | Lucens | AB120541 | AB196945 | AB250116 | AB250135 |
| *P. perfoliatus* L. | Perfoliatus | AB120549 | AB196951 | AB250117 | AB250136 |
| *P. praelongus* Wulf. | Perfoliatus | AB120556 | AB196952 | AB250118 | AB250137 |
| *P. crispus* L. | Crispus | AB120542 | AB196847 | AB250119 | AB250138 |
| *P. maackianus* A. Benn. | Robbinsii | AB120550 | AB196944 | AB250120 | AB250139 |
| *P. compressus* L. | Compressus | AB120558 | AB196846 | AB250121 | AB250140 |
| *P. oxyphyllus* Miq. | Compressus | AB120543 | AB196949 | AB250122 | AB250141 |
| *P. pusillus* L. | Pusillus | AB120547 | AB250148 | AB250123 | AB250142 |
| *P. panormitanus* Biv. | Pusillus | AB120546 | AB196950 | AB250124 | AB250143 |
| *P. obtusifolius*Mert. et Koch | Pusillus | AB120544 | AB196947 | AB250125 | AB250144 |
| *P. octandrus* Poir. | Octandrus | AB120560 | AB196948 | AB250126 | AB250145 |
| *P. cristatus* Regel et Maack | Octandrus | AB120545 | AB196939 | AB250127 | AB250146 |
| *Stuckenia pectinata* (L.) Börner | Pectinatus | AB120562 | AB196953 | AB250128 | AB250147 |

^a^ Species group designated by Wiegleb (1988).
