# Supplementary material for: Molecular Adaptation of rbcL in the Heterophyllous Aquatic Plant Potamogeton
Source: PLoS One. 2009 Feb 27;4(2):e4633. doi: 10.1371/journal.pone.0004633 (PMC2646136; doi:10.1371/journal.pone.0004633)
Supplement: Table S2 — List of primers used for PCR. (0.03 MB DOC) [file pone.0004633.s002.doc]

**Table S2** List of primers used for PCR.

| Gene | Name | Sequence (5'-3') |
| --- | --- | --- |
| *rbcL* | rbcL26F | TGTCACCACAAACAGAGACTAAAGC |
|  | rbcL637F | GAACGTAAACTCACAACCA TTTATG |
|  | rbcL647R | AGCTTCGGCACAAAATAA GAAACGA |
|  | rbcL1375R | ATACG ATCTCTTTCCATACTTCAC |
| *atpB* | atpB23F | TGAGAATCAATCCTACTACTTC |
|  | atpB632F | CGGACTCGTGAAGGAAATGA |
|  | atpB796R | GATGAATAGAAGCACGTCT TG |
|  | atpE1848R | TCCTAGCTCGTCTGAGAGC |
| *petA* | cemA674F | TCTCCTTCGCTT GTAGTCATTTATC |
|  | petA1116F | ATGTGCCAATTGCCATTTAGC |
|  | petA1512R | TTACT YTTGCTACCATCAGGATA |
|  | petA1848R | TTGAACCTTYTCAAACTGTTTCTT |
