# Supplementary material for: Molecular Adaptation of rbcL in the Heterophyllous Aquatic Plant Potamogeton
Source: PLoS One. 2009 Feb 27;4(2):e4633. doi: 10.1371/journal.pone.0004633 (PMC2646136; doi:10.1371/journal.pone.0004633)
Supplement: Table S3 — Parameter estimates and log-likelihood values for Potamogeton atpB under eight codon substitution models included in PAML. (0.05 MB DOC) [file pone.0004633.s003.doc]

**Table S3** Parameter estimates and log-likelihood values for *Potamogeton* *atpB* under

eight codon substitution models included in PAML.

| Model | Log-likelihood | Parameters ^a^ |
| --- | --- | --- |
| **Site-specific models** |  |  |
| M0 : one ω | -2357.5 | ω = 0.098 |
| M1A : nearly neutral | -2348.4 | *p_0_*^a^ = 0.918*,* ω_0_ = 0.000 |
|  |  | *p_1_* = 0.082*,* ω_1_ = 1.000 |
| M2A : positive selection | -2345.1 | *p_0_* = 0.986*,* ω_0_ = 0.036 |
|  |  | *p_1_* = 0.000*,* ω_1_ = 1.000 |
|  |  | *p_2_* = 0.0146*,* ω_2_ = 6.697 |
| M7 : beta | -2348.6 | *p* = 0.005, *q* = 0.049 |
| M8 : beta & ω_s_≥ 1 | -2345.1 | *p_0_* = 0.986 |
|  |  | *p* = 3.719, *q* = 99.000 |
|  |  | *p_1_* = 0.014, ω_1_ = 6.692 |
| M8A : beta & ω_s_=1 | -2348.4 | *p_0_* = 0.918 |
|  |  | *p* = 0.005, *q* = 2.485 |
| **Branch-site models** |  |  |
| Foreground: Heterophylly |  |  |
| Model A (ω_2_ = 1 fixed) | -2348.4 | *p_0_* = 0.918, ω_0_ = 0.000 |
|  |  | *p_1_* = 0.082, ω_1_ = 1.000 |
|  |  | *p_2 +_ p_3_* = 0.000 , ω_2_ = 1.000 |
| Model A (ω_2_ estimated) | -2348.4 | *p_0_* = 0.920, ω_0_ = 0.000 |
|  |  | *p_1_* = 0.082, ω_1_ = 1.000 |
|  |  | *p_2 +_ p_3_* = 0.000 , ω_2_ = 6.692 |
| Foreground: Homophylly |  |  |
| Model A (ω_2_ = 1 fixed) | -2348.4 | *p_0_* = 0.918, ω_0_ = 0.000 |
|  |  | *p_1_* = 0.082, ω_1_ = 1.000 |
|  |  | *p_2 +_ p_3_* = 0.000 , ω_2_ = 1.000 |
| Model A (ω_2_ estimated) | -2348.4 | *p_0_* = 0.920, ω_0_ = 0.000 |
|  |  | *p_1_* = 0.082, ω_1_ = 1.000 |
|  |  | *p_2 +_ p_3_* = 0.000 , ω_2_ = 2.000 |

^a^ the proportion (*p_i_*) of codon sites with ω_i_. In models M7, M8 and M8A, ω was drawn

from a beta distribution B(*p*, *q*) for a proportion (*p_0_*) of sites.
