# Supplementary material for: Molecular Adaptation of rbcL in the Heterophyllous Aquatic Plant Potamogeton
Source: PLoS One. 2009 Feb 27;4(2):e4633. doi: 10.1371/journal.pone.0004633 (PMC2646136; doi:10.1371/journal.pone.0004633)
Supplement: Table S4 — Parameter estimates and log-likelihood values for Potamogeton petA under eight codon substitution models included in PAML. (0.05 MB DOC) [file pone.0004633.s004.doc]

**Table S4** Parameter estimates and log-likelihood values for *Potamogeton* *petA*

under eight codon substitution models included in PAML.

| Model | Log-likelihood | Parameters ^a^ |
| --- | --- | --- |
| **Site-specific models** |  |  |
| M0 : one ω | -1467.7 | ω = 0.174 |
| M1A : nearly neutral | -1462.6 | *p_0_*^a^ = 0.856*,* ω_0_ = 0.000 |
|  |  | *p_1_* = 0.144*,* ω_1_ = 1.000 |
| M2A : positive selection | -1458.0 | *p_0_* = 0.996*,* ω_0_ = 0.123 |
|  |  | *p_1_* = 0.000*,* ω_1_ = 1.000 |
|  |  | *p_2_* = 0.004*,* ω_2_ = 21.287 |
| M7 : beta | -1462.7 | *p* = 0.014, *q* = 0.087 |
| M8 : beta & ω_s_≥ 1 | -1458.0 | *p_0_* = 0.996 |
|  |  | *p* =13.934, *q* = 99.000 |
|  |  | *p_1_* = 0.004, ω_1_ = 21.295 |
| M8A : beta & ω_s_=1 | -1462.6 | *p_0_* = 0.856 |
|  |  | *p* = 0.005, *q* = 2.195 |
| **Branch-site models** |  |  |
| Foreground: Heterophylly |  |  |
| Model A (ω_2_ = 1 fixed) | -1462.6 | *p_0_* = 0.856, ω_0_ = 0.000 |
|  |  | *p_1_* = 0.144, ω_1_ = 1.000 |
|  |  | *p_2 +_ p_3_* = 0.000 , ω_2_ = 1.000 |
| Model A (ω_2_ estimated) | -1462.6 | *p_0_* = 0.856, ω_0_ = 0.000 |
|  |  | *p_1_* = 0.144, ω_1_ = 1.000 |
|  |  | *p_2 +_ p_3_* = 0.000 , ω_2_ = 21.295 |
| Foreground: Homophylly |  |  |
| Model A (ω_2_ = 1 fixed) | -1462.6 | *p_0_* = 0.856, ω_0_ = 0.000 |
|  |  | *p_1_* = 0.144, ω_1_ = 1.000 |
|  |  | *p_2 +_ p_3_* = 0.000 , ω_2_ = 1.000 |
| Model A (ω_2_ estimated) | -1462.6 | *p_0_* = 0.856, ω_0_ = 0.000 |
|  |  | *p_1_* = 0.144, ω_1_ = 1.000 |
|  |  | *p_2 +_ p_3_* = 0.000 , ω_2_ = 2.000 |

^a^ the proportion (*p_i_*) of codon sites with ω_i_. In models M7, M8 and M8A, ω was

drawn from a beta distribution B(*p*, *q*) for a proportion (*p_0_*) of sites.
