# Supplementary material for: Molecular Adaptation of rbcL in the Heterophyllous Aquatic Plant Potamogeton
Source: PLoS One. 2009 Feb 27;4(2):e4633. doi: 10.1371/journal.pone.0004633 (PMC2646136; doi:10.1371/journal.pone.0004633)
Supplement: Table S5 — LRT statistics for testing the hypothesis of positive selection in Potamogeton chloroplast genes. (0.05 MB DOC) [file pone.0004633.s005.doc]

**Table S5** LRT statistics for testing the hypothesis of positive selection in *Potamogeton*

**chloroplast genes.**

| Test No. | Test model | Null model | df |  | Gene | 2ΔL^b^ |
| --- | --- | --- | --- | --- | --- | --- |
| Test 1 | M2A | M1A | 2 |  | *atpB* | 6.5* |
|  |  |  |  |  | *petA* | 9.1* |
| Test 2 | M8 | M7 | 2 |  | *atpB* | 6.9* |
|  |  |  |  |  | *petA* | 9.4** |
| Test 3 | M8 | M8A | -^a^ |  | *atpB* | 6.5** |
|  |  |  |  |  | *petA* | 9.1** |
| Test 4 | Branch-site model A | Branch-site model A |  |  |  |  |
|  | (ω_2_ estimated) | (ω_2_ =1 fixed) |  |  |  |  |
|  |  | Foreground: Heterophylly | 1 |  | *atpB* | n.s. |
|  |  |  |  |  | *petA* | n.s. |
|  |  | Foreground: Homophylly | 1 |  | *atpB* | n.s. |
|  |  |  |  |  | *petA* | n.s. |

^a^ The test statistic for the M8A-M8 comparison is compared with 50:50 mixture of df = 0 and 1

according to Swansson et al. (2003).

^b^ *: *p* < 0.05, **: *p* < 0.01, n.s.: not significant
