# Supplementary material for: Molecular Adaptation of rbcL in the Heterophyllous Aquatic Plant Potamogeton
Source: PLoS One. 2009 Feb 27;4(2):e4633. doi: 10.1371/journal.pone.0004633 (PMC2646136; doi:10.1371/journal.pone.0004633)
Supplement: Text S1 — Abstract in Japanese. (0.03 MB DOC) [file pone.0004633.s006.doc]

**Text S1** Abstract in Japanese.

**研究の背景**水生植物は異形葉形成とよばれる著しい表現型可塑性を示す。異形葉形成種（heterophyllous species）の沈水葉、浮葉、陸生葉といった各葉型の外部形態や解剖学的特徴は、周りの環境にうまく適応している。一方で、異形葉非形成種（homophyllous species）は、沈水葉のみを形成し水面下で生育する。従来これらの形質の進化が、種に固有の光合成活性を最適化しようとする自然選択圧の存在下で、どのような進化的帰結を伴ってきたのかについては、明らかになっていない。最近の進化学的解析では、ルビスコの触媒サブユニットをコードする葉緑体遺伝子*rbcL*は、大部分の陸上植物の系統で正の選択圧を受け進化したことが示された。私たちは水生植物における異形葉の獲得と喪失に関連する適応進化の過程を解明するため、生態的に多様な種を含む日本産ヒルムシロ属*Potamogeton*の*rbcL*遺伝子に対する正の自然選択について解析した。

**主な結果**　　系統解析と最尤法を用いたコドン置換モデルは、日本産ヒルムシロ属の*rbcL*が正の自然選択をうけて進化してきたことを示した。正の自然選択は異形葉形成系統で検出されたが、非形成系統では検出されず、より強い自然選択圧が前者に作用していた。日本産ヒルムシロ属の12箇所のアミノ酸置換は、RbcLの立体構造において重要な部位に位置しており、そのうち部位225と281の２箇所は、直接正の自然選択をうけた部位であると推定された。

**結論／重要性**私たちの解析では、アミノ酸置換と異形葉形成状態との間に完全な相関は見いだせなかった。しかしヒルムシロ属では、特定の系統の*rbcL*にのみ正の自然選択が働いたことを発見した。このことは光合成系に対して、異形葉形成種と非形成種がさらされている対照的な環境条件が異なる自然選択圧として作用したことを意味する。RbcLのアミノ酸置換の増大は、変化する環境条件に対して、絶えずルビスコ構造の微調整が行われていることを反映しているのだろう。
